# Importance of Human Leukocyte Antigen (HLA) Class I and II Alleles on the Risk of Multiple Sclerosis

**DOI:** 10.1371/journal.pone.0036779

**Published:** 2012-05-07

**Authors:** Jenny Link, Ingrid Kockum, Åslaug R. Lorentzen, Benedicte A. Lie, Elisabeth G. Celius, Helga Westerlind, Marie Schaffer, Lars Alfredsson, Tomas Olsson, Boel Brynedal, Hanne F. Harbo, Jan Hillert

**Affiliations:** 1 Department of Clinical Neuroscience, Karolinska Institutet, Stockholm, Sweden; 2 Institute of Immunology, Oslo University Hospital, Rikshospitalet, Oslo, Norway; 3 Department of Neurology, Oslo University Hospital, Ullevål, Oslo, Norway; 4 Department of Medical Genetics, University of Oslo, Oslo, Norway; 5 Department of Laboratory Medicine, Karolinska Institutet, Stockholm, Sweden; 6 Institute of Environmental Medicine, Karolinska Institutet, Stockholm, Sweden; 7 Institute of Clinical Medicine, University of Oslo, Oslo, Norway; Seoul National University College of Medicine, Republic of Korea

## Abstract

Multiple sclerosis (MS) is a complex disease of the central nervous system of unknown etiology. The human leukocyte antigen (HLA) locus on chromosome 6 confers a considerable part of the susceptibility to MS, and the most important factor is the class II allele HLA-DRB1*15:01. In addition, we and others have previously established a protective effect of HLA-A*02. Here, we genotyped 1,784 patients and 1,660 healthy controls from Scandinavia for the *HLA-A*, *HLA-B*, *HLA-C* and *HLA-DRB1* genes and investigated their effects on MS risk by logistic regression. Several allele groups were found to exert effects independently of DRB1*15 and A*02, in particular DRB1*01 (OR = 0.82, p = 0.034) and B*12 (including B*44/45, OR = 0.76, p = 0.0028), confirming previous reports. Furthermore, we observed interaction between allele groups: DRB1*15 and DRB1*01 (multiplicative: OR = 0.54, p = 0.0041; additive: AP = 0.47, p = 4×10^−06^), DRB1*15 and C*12 (multiplicative: OR = 0.37, p = 0.00035; additive: AP = 0.58, p = 2.6×10^−05^), indicating that the effect size of these allele groups varies when taking DRB1*15 into account. Analysis of inferred haplotypes showed that almost all DRB1*15 bearing haplotypes were risk haplotypes, and that all A*02 bearing haplotypes were protective as long as they did not carry DRB1*15. In contrast, we found one class I haplotype, carrying A*02-C*05-B*12, which abolished the risk of DRB1*15. In conclusion, these results confirms a complex role of HLA class I and II genes that goes beyond DRB1*15 and A*02, in particular by including all three classical HLA class I genes as well as functional interactions between DRB1*15 and several alleles of *DRB1* and class I genes.

## Introduction

In the past few years, the number of genes known to influence the risk of multiple sclerosis (MS, OMIM:126200), a chronic inflammatory disease of the central nervous system, has increased dramatically. Even so, the by far most strongly acting genetic signal originates from the human leukocyte antigen (HLA) gene complex on the short arm of chromosome 6. The discovery of the class II risk haplotype, later shown to be best represented by the HLA-DRB1*15:01 allele, was made almost 40 years ago and was only recently shown to be accompanied by an independent protective allele group in class I, HLA-A*02 [1], [2], [3], [4], [5], [6]. In addition to these two established alleles a series of reports have suggested additional MS risk alleles as well as interactions between alleles [7]. Within the *HLA-DRB1* gene, the allele groups DRB1*03, DRB1*14, DRB1*07 and DRB1*11 have all been suggested to influence risk in MS [8], [9], [10], [11]. Furthermore, interactions have been implicated among *DRB1* allele groups: DRB1*08, DRB1*10 and DRB1*01 were reported to interact with DRB1*15 in modifying susceptibility to MS [8], [10].

An increasing number of studies has recently confirmed the importance of genes in the class I region on MS susceptibility [12], [13]. The HLA-A*02 association was first identified in a limited Swedish cohort [2] and was later confirmed in a larger Scandinavian cohort [1]. In addition, studies including not only *HLA-DRB1* and *HLA-A*, but also *HLA-B* and/or *HLA-C* have indicated additionally associated alleles [14], [15], [16], [17], including HLA-C*08, HLA-C*05 and HLA-B*44. A single nucleotide polymorphism (SNP) genotyping approach, where HLA alleles were imputed based on linkage disequilibrium with SNP markers, found a signal of association from HLA-B*44:02 when conditioning on DRB1*15 [18]. HLA-B*44 was also found associated independently from DRB1*15 in a meta-analysis of available SNP genome scans [19]. The *MOG* gene, encoding the myelin oligodendrocyte glycoprotein and positioned telomeric of *HLA-A*, has been repeatedly studied [20], [21] but its signal of association seems most likely secondary to HLA-A*02 [15].

Several studies have tried to distinguish which of the class I alleles or genes that are responsible for the class I region signal. When carried together, HLA-C*05 was reported to enhance the protective effect of HLA-A*02 [15], but this could not be replicated in the Scandinavian population [17]. HLA-C*05 has been seen to be secondary to both HLA-A*02 and HLA-B*44 whereas B*44 was not secondary to HLA-A*02 [22]. In addition to this, a SNP screen revealed a SNP associated to MS in the untranslated region of *HLA-G* that could not be ruled out to be independent from either *HLA-A*, -*B* or -*C* alleles [23].

In this study, we aimed at assessing signals from the three classical class I genes together with *HLA-DRB1* in a large cohort consisting of Swedish and Norwegian MS patients and controls. All three major genes in class I as well as *HLA-DRB1* have been genotyped with robust direct HLA genotyping techniques and the association with MS and potential interactions were assessed with logistic regression.

## Results

The aim of this study was to investigate the association to MS of all three classical HLA class I genes, *HLA-A*, *HLA-B*, *HLA-C* as well as the HLA class II gene, *HLA-DRB1*, in a Scandinavian cohort of 1,784 patients and 1,660 controls. The nature of the previously reported effects within class I in MS was also evaluated.

### HLA class I and II allele groups in MS risk

We started by assessing the mode of inheritance of the two already established genetic factors within the HLA region to determine how to proceed with the analysis. HLA-DRB1*15 was seen to behave multiplicatively as the odds ratio for carrying one copy of DRB1*15 (95% confidence interval) was 3.03 (2.62–3.52) and 11.47 (7.74–16.99) for carrying two copies. In contrast HLA-A*02 behaved dominantly, the odds ratio for carrying one copy of A*02 was 0.70 (0.61–0.81) and 0.57 (0.45–0.71) for carrying two copies. Therefore these particular allele groups were encoded accordingly throughout this study (see material and methods section for details).

There was no difference between coding the additional allele groups as gene dose (0, 1 or2) or carriage (0 or 1) when assessing which allele groups should be in the final regression model (data not shown), but the overall fit of the model (deviance) was superior in the dominant model. Therefore only carriage coded results will be presented. In the final model DRB1*15 and B*14 were positively associated with MS while A*02, B*12, DRB1*01 and DRB1*07 were negatively associated (Table 1). Thus, *HLA-B* seemed to influence MS risk in addition to *HLA-A* and *HLA-DRB1* but *HLA-C* was not independently associated with MS at this stage.

**Table 1:** Risks carried by each of the nominally significant allele groups from the final logistic regression analyses.

Allele group	p-value (nominal)	p-value (FDR)	OR (95% C.I.)	Frequency Cases (%)	Frequency Controls (%)
DRB1*15 (dose)	2.51×10^−63^	2.26×10^−62^	3.03 (2.67–3.46)	35.4	15.3
A*02	5.42×10^−08^	2.44×10^−07^	0.67 (0.58–0.77)	27.7	34.6
B*14	6.96×10^−05^	0.00013	2.05 (1.45–2.94)	2.7	1.8
DRB1*07	0.0016	0.0020	0.70 (0.56–0.87)	5.0	8.5
B*12	0.0025	0.0028	0.76 (0.63–0.91)	8.8	13.1
DRB1*01	0.034	0.034	0.82 (0.68–0.98)	7.7	11.9

FDR = False discovery rate, performed in R, based on the paper [37].

Next, we wanted to assess whether groups stratified by DRB1*15 carriage display different genetic etiology. 1,051 cases and 476 controls were DRB1*15 positive while 733 cases and 1,184 controls were negative for DRB1*15 in this cohort. Allele group frequencies are shown in Table S1. Six allele groups reached significance in the HLA-DRB1*15 negative group whereas four allele groups reached significance in the DRB1*15 positive group (Table 2). Surprisingly, apart from A*02, which was negatively associated in both strata, the other allele groups were different between the groups. In the negative group we found associations to B*14, A*02, DRB1*07, B*12, A*10 and B*18 and in the positive group both DRB1*15 and A*02 as well as C*12 and DRB1*01 were associated (Table 2). An importance of DRB1*01 in DRB1*15 positives has previously been reported [8] and was thus confirmed. In addition, even allele groups of *HLA-C* may influence the risk of MS, at least in the DRB1*15 positive individuals.

**Table 2:** Risk carried by each of the nominally significant allele groups in the logistic regression model for carriage coded DRB1*15 negative and positive strata.

DRB1*15 negative					
Allele group	p-value (nominal)	p-value (FDR)	OR (95% C.I.)	Frequency Cases (%)	Frequency Controls (%)
B*14	0.00040	0.0018	2.08 (1.39–3.13)	4.0	2.2
A*02	0.00086	0.0026	0.72 (0.59–0.87)	27.8	34.7
DRB1*07	0.0032	0.0058	0.66 (0.5–0.87)	6.8	9.9
B*12	0.0045	0.0068	0.71 (0.56–0.9)	9.3	14.2
A*10	0.012	0.012	1.52 (1.09–2.1)	6.3	3.7
B*18	0.011	0.012	1.69 (1.13–2.54)	4.0	2.2

DRB1*15 positive					
Allele group	p-value (nominal)	p-value (FDR)	OR (95% C.I.)	Frequency Cases (%)	Frequency Controls (%)
DRB1*15	7.99×10^−11^	5.60×10^−11^	3.87 (2.61–5.92)	60.1	53.2
A*02	3.31×10-05	0.00012	0.62 (0.49–0.78)	27.6	34.2
C*12	0.00021	0.00050	0.5 (0.35–0.72)	4.1	6.6
DRB1*01	0.0052	0.0052	0.59 (0.41–0.86)	3.6	6.3

Country of origin and gender were included in the models as covariates. DRB1*15 was coded as dose (0,1,2), all other allele groups were coded for carriage (0,1).

The difference in association pattern between the DRB1*15 positive and negative subpopulation prompted us to formally assess the presence of interaction. The allele groups DRB1*07, B*12, B*18, A*10 and B*14, failed to show a significant interaction with DRB1*15. However two allele groups (C*12 and DRB1*01) did reach significant statistical interaction when using either departure from multiplicativity as definition, or when analyzed by attributable proportion due to interaction (AP) (i.e. departure from additivity) (Table 3). In addition, A*02 showed statistically significant interaction with DRB1*15 in AP analysis only.

**Table 3:** Interaction analysis showing evidence of interaction between *DRB1* * *15* and other allele groups influencing the risk of MS.

	Logstic regression, statistical interaction					Attributable proportion, biologic interaction		
Interaction term	OR *DRB1* * *15*	OR interacting allele	OR (interaction term, 95% C.I.)	nominal p-value (interaction term)	Bonferroni corrected p-value	AP (95% C.I.)	nominal p-value	Bonferroni corrected p-value
DRB1*15×C*12#	3.84	1.6	0.37 (0.21–0.64)	0.00035	0.0028	0.58 (0.31–0.84)*	2.57×10-05	0.00021
DRB1*15×DRB1*01#	3.74	0.97	0.54 (0.36–0.82)	0.0041	0.033	0.47 (0.27–0.66)*	4.00×10-06	3.20×10-05
DRB1*15×A*02	3.54	0.66	1.01 (0.76–1.35)	0.92	1.00	0.23 (0.067–0.40)*	0.0058	0.046
DRB1*15×B*18#	3.66	1.97	0.54 (0.32–0.91)	0.019	0.15	−0.23 (−0.68–0.23)	0.33	1.00
DRB1*15×A*10	3.7	1.79	0.63 (0.39–1.00)	0.049	0.39	−0.049 (−0.43–0.34)	0.80	1.00
DRB1*15×B*12	3.35	0.61	1.25 (0.87–1.79)	0.23	1.00	0.12 (−0.11–0.35)*	0.29	1.00
DRB1*15×DRB1*07	3.38	0.64	1.24 (0.78–1.98)	0.36	1.00	0.1 (−0.21–0.41)*	0.53	1.00
DRB1*15×B*14	3.63	2.02	1.00 (0.45–3.00)	1.00	1.00	0.36 (−0.13–0.84)	0.15	1.00

* = risk is coded as absence of this allele group since it is nominally protective.

# = Model has significantly better fit than one with only the two variables by themselves (no interaction term).

In conclusion, we observed significant support for interaction for MS risk between HLA class I and class II genes as well as between allele groups of *HLA-DRB1* itself. This confirms and extends previous reports of such interactions [8].

### Evaluation of protective effects of class I allele groups in MS

Previous reports have indicated HLA-C*05 and HLA-B*44 as important allele groups in MS, and Healy et al used logistic regression to study these allele groups with the previously established HLA-DRB1*15 and HLA-A*02 as covariates [22]. It has also been reported that the protective effect of A*02 is enhanced when also carrying C*05 in the Italian population [14]. In a previous study, we failed to confirm this role of C*05 [17]. Now, when being able to study also B*12, the protective effect of HLA-C*05 was seen to be secondary to both B*12 and HLA-A*02 due to linkage disequilibrium (Table S2). Again, we failed to confirm an augmented protective effect of A*02 when carrying C*05 (A*02 alone OR = 0.66, p = 5.95×10^−09^; carrying A*02 and C*05 OR = 0.64, p = 2.65×10^−04^).

In recent years, SNP genotyping has widely been applied in attempts to map genetic effects within the HLA gene region. Early attempts failed to find much residual effects after stratifying for DRB1*15 [13]. More recently, Cree et al reported an association to the SNP rs4959039 (A/G), located in the downstream untranslated region of *HLA-G* [23]. This SNP was found to be associated independently of a DRB1*15 tagging SNP but it could not be determined if it tagged HLA-A*02 or was independent of this allele group. We genotyped this marker in 1,978 patients and 1,782 controls previously genotyped for *HLA-A* and *HLA-DRB1* and observed a minor allele frequency (G) of 0.26 in cases and 0.33 in controls. A haplotype analysis was performed using UNPHASED to elucidate the correlation between rs4959039 and *HLA-A*. The correlation between minor allele rs4959039*^G^* and HLA-A*02 was clearly higher compared to the other top five *HLA-A* allele groups with a D′ of 0.90 and r^2^ of 0.78. There was no residual association with the SNP when correcting for A*02 (p = 0.90) in logistic regression, convincingly showing that the rs4959039 effect was secondary to A*02.

### Haplotype analysis

Allele group association analysis assesses the combined risk of all the haplotypes carrying certain allele groups. The effects of associated HLA allele groups in logistic regression are independent of each other but a shown risk or protection can, alternatively, indicate a stronger effect at the haplotype level. The property of the HLA gene complex to harbor these extended conserved haplotypes often spanning several megabases, may be seen as an argument against case-control design where phase cannot be deduced. To compensate for this void, we attempted to infer haplotypes using UNPHASED 3.0.13, hypothesizing that the greater available numbers in this case control study compared with available family cohorts could outweigh the disadvantages. Table 4 contains the 20 most frequent inferred haplotypes from this analysis. Only haplotypes with a probability of at least 80% were used in the association analysis. Similar results were obtained when all haplotypes, irrespectively of probability, were included (data not shown).

**Table 4:** Frequencies of estimated haplotypes, odds ratios and p-values from logistic regression with the 20 most common haplotypes.

	Haplotype											
No.	HLA-A	HLA-C	HLA-B	HLA-DRB1	Cases	Controls	Frequency Cases (%)	Frequency Controls (%)	Frequency Total (%)	Nominal p-value	FDR corrected p-value	Odds Ratio (95% CI)
1.	1	7	8	3	207	240	7.0	8.6	7.7	0.022	0.033	0.78 (0.63–0.96)
2.	3	7	7	15	226	118	7.6	4.2	6.0	2.97×10^−07^	2.28×10^−06^	1.90 (1.49–2.44)
3.	2	7	7	15	179	59	6.0	2.1	4.1	1.18×10^−12^	2.72×10^−11^	3.14 (2.31–4.34)
4.	2	3	15	4	53	86	1.8	3.1	2.4	0.0066	0.015	0.60 (0.42–0.87)
5.	3	4	35	1	42	72	1.4	2.6	2.0	0.0030	0.0087	0.54 (0.36–0.81)
6.	2	5	12	4	25	81	0.8	2.9	1.8	1.39×10^−07^	1.60×10^−06^	0.28 (0.17–0.45)
7.	9	7	7	15	78	23	2.6	0.8	1.7	1.45×10^−06^	8.33×10^−06^	3.30 (2.06–5.47)
8.	2	3	40	6	38	56	1.3	2.0	1.6	0.068	0.097	0.67 (0.43–1.03)
9.	10	12	18	15	44	22	1.5	0.8	1.1	0.011	0.021	2.00 (1.19–3.48)
10.	3	3	15	4	33	15	1.1	0.5	0.8	0.0078	0.016	2.35 (1.27–4.52)
11.	1	6	37	15	37	10	1.2	0.4	0.8	0.00021	0.00095	3.87 (1.97–8.35)
12.	2	5	12	15	24	23	0.8	0.8	0.8	0.80	0.82	0.92 (0.51–1.69)
13.	1	7	7	15	30	15	1.0	0.5	0.8	0.020	0.032	2.15 (1.15–4.19)
14.	11	4	35	1	22	21	0.7	0.8	0.7	0.59	0.68	1.19 (0.63–2.23)
15.	19	16	12	7	21	22	0.7	0.8	0.7	0.82	0.82	0.93 (0.49–1.74)
16.	19	3	40	4	12	31	0.4	1.1	0.7	0.0063	0.015	0.38 (0.18–0.74)
17.	2	7	8	3	16	26	0.5	0.9	0.7	0.076	0.10	0.56 (0.29–1.05)
18.	2	3	40	4	17	24	0.6	0.9	0.7	0.20	0.26	0.65 (0.34–1.25)
19.	2	3	15	6	16	24	0.5	0.9	0.7	0.23	0.28	0.67 (0.34–1.27)
20.	3	7	7	4	16	18	0.5	0.6	0.6	0.66	0.72	0.85 (0.42–1.71)

The haplotype analysis revealed several nominally significant associated haplotypes in spite of low frequencies. Interestingly, almost all of the most common haplotypes contain one or more allele groups found to be associated in the final full logistic regression model (Table 1). A striking finding was that almost all DRB1*15 carrying haplotypes in Table 4 were risk haplotypes and all A*02 carrying haplotypes were protective as long as they did not carry DRB1*15. There was however an interesting exception (haplotype number 12 in Table 4) where A*02 together with C*05 and B*12 abolished the risk of DRB1*15. To further investigate these allele groups, logistic regressions with the 20 most common haplotypes carrying each of these allele groups was created (Table S3, S4, S5, S6). Indeed almost all DRB1*15 haplotypes were risk haplotypes even though not all reached significance for association with MS (Table S3). The exception was again the haplotype carrying A*02, C*05 and B*12, OR (95% C.I.) = 1.13 (0.62–2.07) and p-value = 0.71 thus somewhat lowering the risk of DRB1*15. The same analysis was done for A*02 bearing haplotypes (Table S4), and also here the pattern from Table 4 was repeated. Most of the B*12 haplotypes were protective unless carrying DRB1*15 or DRB1*06 (Table S5). When looking at the C*05 bearing haplotypes, C*05 was only seen in haplotypes with either B*12, B*18 or B*05 and varied in risk for MS (Table S6). One should bear in mind that these haplotypes were indeed estimated and the haplotype frequencies were small.

The protective haplotype carrying A*02, C*05 and B*12 (hereafter referred to as “protective class I haplotype” or abbreviated as “H”) was further studied for the nature of its protective properties in relation to DRB1*15 and if any of its alleles were sole responsible for the protective effect. The protective class I haplotype was independent of DRB1*15 and was protective on both DRB1*15 positive and DRB1*15 negative haplotypes (Table 5).

**Table 5:** Independence of the protective class I haplotype from *DRB1* * *15* demonstrated in two ways.

	Haplotype +		Haplotype -			
	Cases	Controls	Cases	Controls	OR (95% CI)	p-value
***DRB1*** * ***15 +***	24	23	1027	409	0.42 (0.23–0.74)*	0.0041
***DRB1*** * ***15 −***	43	122	1880	2244	0.42 (0.30–0.60)*	1.141×10^−06^
**OR (95% CI)**	2.96 (1.52–5.78)**		3.00 (2.63–3.41)**			
**p-value**	0.0021		<2.2×10^−16^			

Counts are made in number of chromosomes carrying the haplotypes estimated in UNPHASED within the cohort. “Haplotype +” was all chromosomes with both A*02, C*05 and B*12 disregarding the *DRB1* locus, “Haplotype –”were all chromosomes that did not have all three allele groups (A*02, C*05 and B*12) at once, still disregarding DRB1. “DRB1*15+” was all chromosomes carrying DRB1*15 disregarding class I allele groups and vice versa for the “DRB1*15 –”.

* ) Odds ratio for the class I haplotype within DRB1*15 positive and negative haplotypes respectively.

** ) Odds ratio for DRB1*15 within the class I haplotype and class I haplotype negative respectively. Note: Class I haplotype negative chromosomes can carry parts of the protective haplotype (i.e. A*02 but in that case not C*05 and/or B*12).

**Figure 1:**
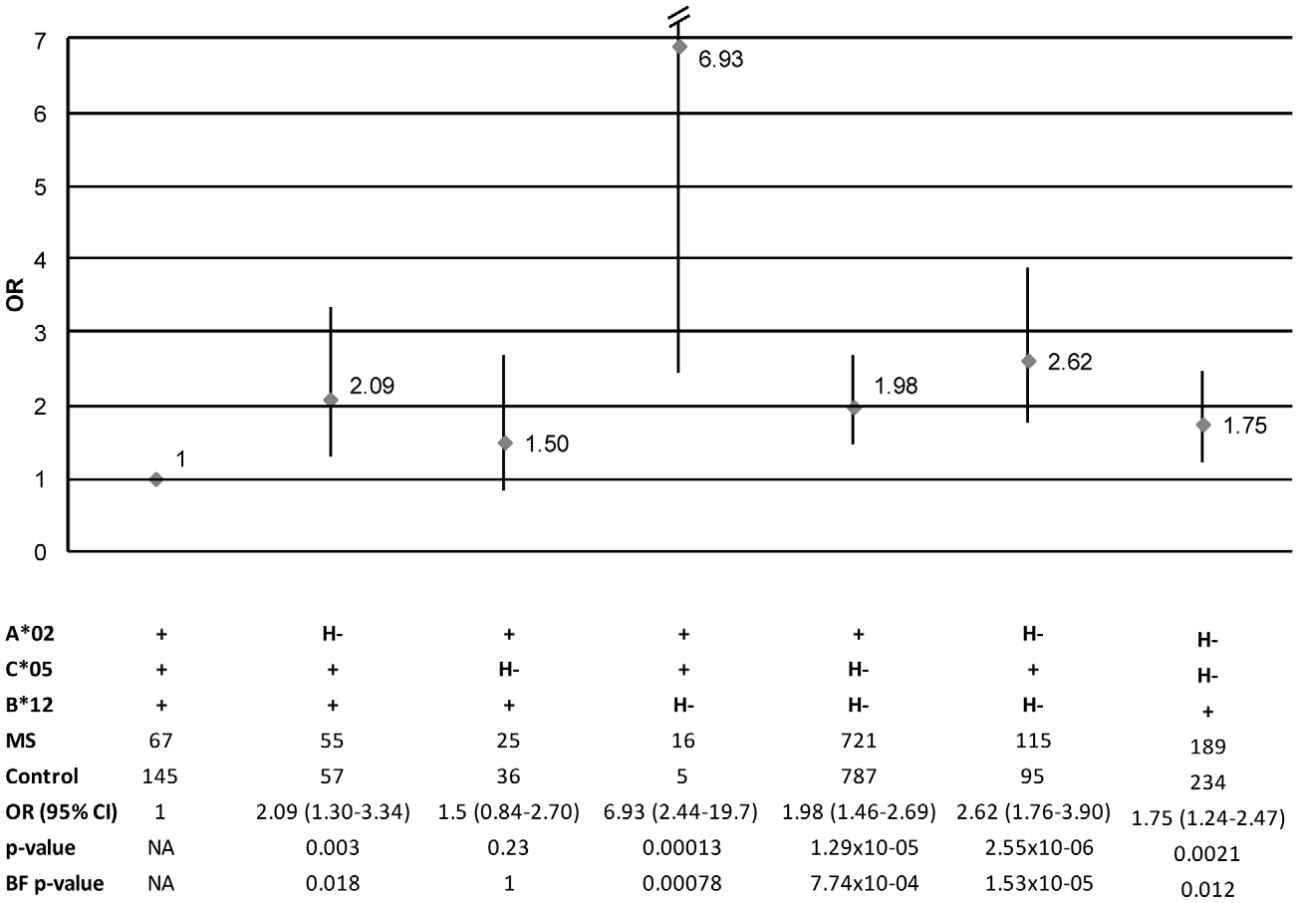
Odds ratios for haplotypes carrying any combination of A*02, C*05 and B*12. Odds ratios for haplotypes containing one or two of the allele groups of the protective class I haplotype compared to the full protective class I haplotype disregarding the *DRB1* locus. Chromosomes carrying both A*02 and B*12 was the only combination within this setting that had the same risk as the protective haplotype, the risk of MS was significantly higher for all other combinations.“+” = Chromosomes carrying the allele group specified to the left, “H−” = chromosomes carrying other allele group than the one specified to the left (i.e. not the complete protective haplotype). OR = odds ratio with 95% confidence interval, nominal p-value and Bonferroni corrected p-values derived from chi square test with full protective haplotype as reference.

Chromosomes positive for both A*02 and B*12 but lacking C*05 was the only combination of the protective class I haplotype that did not have a significantly higher risk than chromosomes carrying all three allele groups (H+)(Figure 1). Thereby indicating that a haplotype containing at least A*02 and B*12 seems responsible for this protective effect in our cohort.

**Figure 2:**
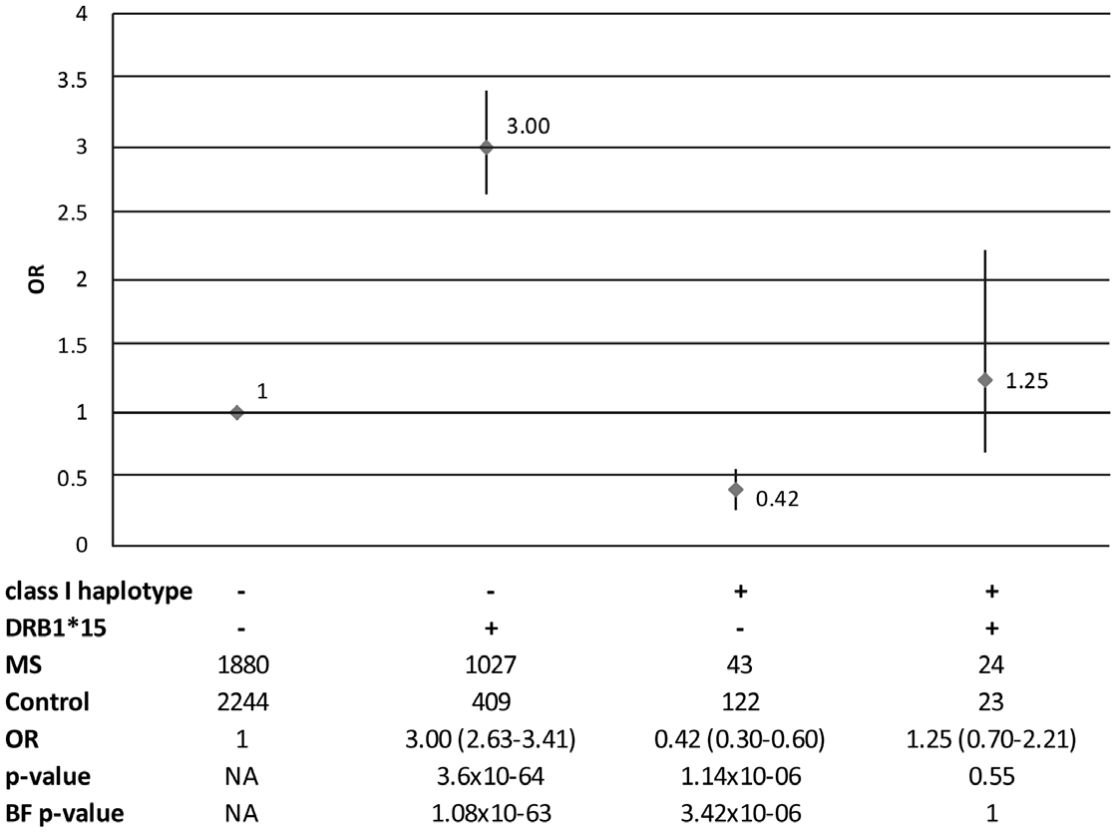
Odds ratios for chromosomes carrying only the protective haplotype or DRB1*15 or both. Chromosomes carrying both DRB1*15 and the protective class I haplotype do not differ in risk of MS from chromosomes not carrying either of them (reference). DRB1*15 carriage increase risk of MS significantly and carriage of the class I haplotype significantly decrease risk of MS. Counts are measured in chromosomes and only individuals with estimated haplotypes with a probability of more than 80% were included. “+” = Chromosomes positive for the protective haplotype or DRB1*15, “−” and “H−” = chromosomes negative for DRB1*15 or the protective haplotype. OR = odds ratio with 95% confidence interval, nominal p-value and Bonferroni corrected p-values derived from chi square test with doubly negative chromosomes as reference.

When studying the effect of the protective class I haplotype in the context of DRB1*15, it was seen that compared to doubly negative chromosomes (“H− DRB1*15-” neither carrying the protective class I haplotype nor DRB1*15), chromosomes carrying DRB1*15 but negative for protective class I haplotype had an OR of 3 (p-value 1.08×10^−63^) (Figure 2). Chromosomes carrying the protective class I haplotype but not DRB1*15 had a negative association compared to doubly negative chromosomes (OR 0.42, p-value 3.42×10^−06^). Chromosomes carrying both A*02, C*05, B*12 and DRB1*15 did not differ in risk from doubly negatives (OR 1.25, p-value 1). Thus, this haplotype diminishes the risk of DRB1*15 also in this analysis.

Analysis of the haplotype was also performed with logistic regression to be able to account for carriage of A*02, C*05 and B*12 as well as baseline covariates as country of origin, gender and carriage of DRB1*15. After including the three class I allele groups and adding carriage of the haplotype into the model both A*02 (OR 0.73 p = 0.00015), B*12 (OR 0.69 p = 0.0032) and the protective haplotype (OR 0.54 p = 0.0080) were significantly associated to MS. Carriage of C*05 was not significant and DRB1*15 was independently associated to MS in the model (OR 3.28 p = <2×10^−16^). The overall fit (deviance) of the model was significantly better than the model with only baseline variables and carriage of the three class I allele groups (p = 0.0076).

## Discussion

The aim of this study was to investigate the class I and class II allele groups and their effect on MS susceptibility in the Scandinavian population. All 3,444 individuals were genotyped for *HLA-A*, *-B*, *-C*, and *-DRB1*.

We have confirmed and extended previous reports of a complex genetic influence of the HLA region on MS susceptibility. The two strongest effects are clearly conferred by DRB1*15 and A*02 but we could also show that additional *DRB1* allele groups contribute to risk as well as allele groups of *HLA-B* which seem more important than those of *HLA-C*. Further, several alleles were interacting with DRB1*15, exerting their effects either in DRB1*15 positive or negative individuals. However, it was clear that no other class I allele group equaled the effect of A*02.

A novelty of this study is the application of formal interaction analysis. We could see that several of the associations from the full analysis persisted in only one of the two DRB1*15 strata. Two HLA allele groups did interact with DRB1*15, namely C*12 and DRB1*01. A protective effect of DRB1*01 was seen both in the full sample set and in the DRB1*15 positives. This *trans* interaction has been discussed previously where an under transmission of DRB1*01 was detected in DRB1*15 positive affected children in family trios [8]. How such an interaction could be explained molecularly is potentially important as it may give clues on how HLA genotype exerts its role on MS risk. Explanations could potentially involve competition for peptides or trans-coded HLA class II heterodimers [24]. The latter mechanism would be more likely to occur at the HLA-DQ locus where both α- and β-chains are polymorphic (in contrast to the almost non-polymorphic DRα-chain).

The interaction between DRB1*15 and A*02 and DRB1*15 and C*12 suggests that they are involved in the same pathway of developing disease but not necessarily exerting their effect at the same time or interact physically since they act mainly in different immune pathways. This data supports the hypothesis that viral infections play an important role in susceptibility to MS.

Traditionally, it has been argued that the existence of extended haplotypes spanning HLA class I and class II indicates a functional property being evolutionarily advantageous and potentially important for the risk of autoimmune disease. As shown in Table 4, all haplotypes were of limited frequency and none of the most common ones showed stronger risk than the most clearly associated allele groups. Haplotypes carrying DRB1*15 were almost always risk haplotypes regardless of which other allele groups that were present, indicating that it is probably the DRB1*15 allele itself that confers the risk of MS. The protective class I haplotype suggests a protection against MS that must be further studied to evaluate two things: 1) is this haplotype tagging some other marker in the HLA or 2) is the protection inflated by combining several independent protective markers that when carried together overcome the effect of DRB1*15.

Recently, the International MS Genetics Consortium (IMSGC) published an analysis of SNP-derived inferred HLA genotypes as part of a genome wide association study on close to 10.000 patients [25]. Also here, a dominance of a DRB1*15 effect was followed by an influence of A*02. In addition, residual effects were explained by other *DRB1* alleles and little further signal in class I was detected which would be in line with our suggestion of the protective class I haplotype. Besides from harboring the classic HLA genes, the HLA gene complex is rich in other genes of importance for immune function. It is plausible that such genes are of importance also in MS but the proximity of the two most strongly acting MS genes is likely to obscure such signals making them difficult to identify. In essence the main result of the IMSGC screen of the HLA gene complex is that the strongest effects indeed map to the classic HLA genes themselves, both for the class II and class I subregions.

The confirmation of an importance for one or even several HLA class I genes in MS, paralleled by findings in other autoimmune diseases [18], points toward a possible importance of class I restricted CD8+ T-cells. Our group has previously studied this in the MS-like model experimental autoimmune encephalomyelitis (EAE) after immunization with myelin basic protein (MBP). Here, protection against disease was found in rats carrying the MHC class I u and MHC class II a haplotype, an effect efficiently abrogated by antibodies against CD8+ cells [26]. A series of studies using double and triple transgenic mice has further illustrated how class I restricted lymphocytes may contribute to MS-like autoimmune events [27], [28]. The explanation could be that a combination of class I allele groups, such as A*02-C*05-B*44, immunologically counteract the negative effect of carrying DRB1*15 in class II when encountering some pathogen involved in MS susceptibility. This mechanism could vary in effect size between populations since the environmental challenge may be different in different areas.

In conclusion, evidence is suggesting that HLA class I and class II effects contribute and interact in determining the risk of MS, as in autoimmune diseases in general. These genes are the most strongly associated with MS and the functional background to these effects should be given increased attention.

## Materials and Methods

### Subjects

All 1784 patients were diagnosed with MS according to the McDonald or Poser criteria [29], [30]. The 1,229 Swedish patients were gathered through the neurology clinics at Karolinska University Hospital, Stockholm, Sweden. The ethical board of Karolinska Institutet approved of the study and oral or written informed consent was obtained from all participants according to the ethical permission. From Norway 555 MS patients were collected from the Oslo MS registry at the Department of Neurology, Ullevål, Oslo University Hospital. The study was approved by the Regional Committee for Medical and Health Research Ethics, and written informed consent was obtained from all individuals.

Of the 1,660 healthy controls used in this study, 1,062 were Swedish blood donors of Nordic origin giving informed consent. From Norway 598 healthy controls were recruited from the Norwegian Bone Marrow Donor Registry, (http://www.nordonor.org/). Age and gender distribution for cases and controls are shown in Table 6.

**Table 6:** Description of basic data concerning the subjects studied.

	Swedish		Norwegian	
	Patients	Controls	Patients	Controls
**Number of subjects**	1229	1062	555	598
**Average age (year range)**	55.1 (25–89)	49.3 (24–79)	57.0 (25–93)	45.1 (29–60)
**Female:Male ratio**	2.5	1.7	2.8	1.9
**% ** DRB1*15 ** carriers**	58.5	29.5	59.8	27.3
**% ** A*02 ** carriers**	46.5	57.3	46.3	54.3

### Methods

HLA genotyping was performed using several techniques. The Swedish cohort was genotyped for *HLA-DRB1, HLA-A* and *HLA-C* using Olerup SSP™ HLA Low resolution Kit [31] and for *HLA-B* using a Luminex based reverse PCR-SSO (LABType® SSO from One Lambda, Inc., Canoga Park, CA, USA). The Norwegian patients were genotyped using PCR with sequencing-based approach [32] and the controls were genotyped with various techniques used in the Norwegian Bone Marrow Donor registry laboratory, (http://www.nordonor.org/).

### SNP genotyping and analysis

Swedish MS cases (N = 2,576) and controls (N = 2,031) were genotyped in the study for correlation analysis between a SNP (rs4959039) in the HLA region and HLA-A*02 status. This group of individuals consisted of the above described patients and controls plus newly diagnosed patients and matched controls from the EIMS study described elsewhere [33]. SNP genotyping was performed using the TaqMan® SNP Genotyping Assays (Applied Biosystems, USA) according to the manufacturer's manual. *HLA-A* and *HLA-DRB1* genotyping was formerly performed using Olerup SSP™ HLA Low resolution Kit [31]. In total, 1,978 patients and 1,782 controls had full information on gender, the SNP, *HLA-A* and *HLA-DRB1* genotypes and were used in the correlation analysis.

### Serological allele groups

As some Norwegian controls were not genotyped down to two digit level, major allele groups were created where there was an information shortage. The allele grouping is shown in Table 7. It should be noted that the B*44 allele group which has previously been reported to be associated to MS is included in the B*12 major allele group together with B*12 and B*45. However, B*44 is the dominating allele within this group (90%).

**Table 7:** Major allele groups and their frequencies in the Scandinavian sample set.

	Allele group frequencies (%)			
Major Allele group	Patients	Controls	Included allele groups (% of total)†	total count less than 150 chromosomes
A*01	14.9	14.3	A*01 (100)	no
A*02	27.7	34.6	A*02 (100)	no
A*03	20.2	16.7	A*03 (100)	no
A*09	11.1	10.0	A*09 (1.9), A*23 (10.9), A*24 (87.2)	no
A*10	6.1	4.3	A*10 (2.8), A*25 (44.8), A*26 (50.6), A*34 (0.3), A*66 (1.7)	no
A*11	5.6	5.4	A*11 (100)	no
A*19	10.3	10.0	A*19 (1.6), A*29 (14.4), A*30 (10.0), A*31 (35.2), A*32 (34.2), A*33 (4.3), A*74 (0.3)	no
A*28	4.1	4.7	A*28 (5.3), A*68 (93.7), A*69 (1.0)	no
C*01	3.6	4.5	C*01 (100)	no
C*02	5.6	5.9	C*02 (100)	no
C*03	17.3	21.0	C*03 (100)	no
C*04	8.1	9.4	C*04 (100)	no
C*05	6.6	8.5	C*05 (100)	no
C*06	6.4	6.9	C*06 (100)	no
C*07	39.6	32.9	C*07 (100)	no
C*08	2.5	2.2	C*08 (100)	no
C*12	3.8	3.3	C*12 (100)	no
C*14	1.1	1.0	C*14 (100)	yes
C*15	3.1	2.2	C*15 (100)	no
C*16	1.5	1.9	C*16 (100)	yes
C*17	0.7	0.5	C*17 (100)	yes
C*18	0.0	0.0	C*18 (100)	yes
B*05	5.4	4.9	B*05 (3.7), B*51 (90.4), B*52 (5.9)	no
B*07	24.3	15.7	B*07 (100)	no
B*08	10.6	12.0	B*08 (100)	no
B*12	8.8	13.1	B*12 (7.1), B*44 (89.5), B*45 (3.5)	no
B*13	0.8	1.6	B*13 (100)	yes
B*14	2.7	1.8	B*14 (98.1), B*64 (0.6), B*65 (1.3)	no
B*15	11.1	11.7	B*15 (83.2), B*62 (16.8)	no
B*16	2.2	2.1	B*16 (6.1), B*38 (26.5), B*39 (67.3)	yes
B*17	2.1	3.2	B*17 (18.2), B*57 (68.5), B*58 (13.3)	no
B*18	5.6	3.3	B*18 (100)	no
B*21	1.1	1.3	B*21 (2.4), B*49 (59.8), B*50 (37.8)	yes
B*22	1.4	1.6	B*22 (5.0), B*55 (44.6), B*56 (50.5)	yes
B*27	5.7	7.2	B*27 (100)	no
B*35	7.0	7.8	B*35 (100)	no
B*37	2.5	1.5	B*37 (100)	yes
B*40	7.5	10.3	B*40 (80.0), B*60 (18.3), B*61 (1.8)	no
B*41	0.7	0.4	B*41 (100)	yes
B*42	0.0	0.0	B*42 (100)	yes
B*47	0.3	0.4	B*47 (100)	yes
B*48	0.1	0.2	B*48 (100)	yes
B*53	0.0	0.0	B*53 (100)	yes
B*67	0.0	0.0	B*67 (100)	yes
B*70	0.0	0.0	B*70 (100)	yes
B*73	0.1	0.0	B*73 (100)	yes
DRB1*01	7.7	11.9	DRB1*01 (100)	no
DRB1*03	10.8	12.7	DRB1*03 (100)	no
DRB1*04	15.9	19.8	DRB1*04 (100)	no
DRB1*05	6.3	8.1	DRB1*05 (1.2), DRB1*11 (77.1), DRB1*12 (21.7)	no
DRB1*06	11.7	15.9	DRB1*06 (1.2), DRB1*13 (86.6), DRB1*14 (12.3)	no
DRB1*07	5.0	8.5	DRB1*07 (100)	no
DRB1*08	5.0	5.0	DRB1*08 (100)	no
DRB1*09	0.9	1.4	DRB1*09 (100)	yes
DRB1*10	0.8	0.8	DRB1*10 (100)	yes
DRB1*15	35.4	15.3	DRB1*15 (99.8), DR2 (0.2)	no
DRB1*16	0.5	0.5	DRB1*16 (100)	yes

Some individuals were not genotyped deeply enough to distinguish allele groups. Therefore, major allele groups containing several allele groups were created when needed.

† Figure in parenthesis is the percentage of this allele group in this major allele group.

### Statistical methods

The software used for statistical calculations in this paper was R version 2.6.2 [34] and UNPHASED 3.0.13 [35]. Nested logistic regression models were compared using analysis of deviance and chi square test in R. In the logistic regressions all allele groups were coded 0, 1, 2 (dose) or 0, 1 (carrier) depending on question and those allele groups with a total count of less than 150 chromosomes (2.2% allele frequency) in the full cohort were grouped within each gene into a group called X (see Table 7). This cutoff was set to decrease complexity of the cohort and increase power. DRB1*08, A*11, B*35 and C*02 were used as intercepts for the full analysis as these were the most evenly distributed between cases and controls and had a frequency of at least 5% in both groups. First, a baseline model including the already known risk allele groups for MS was created. This model included country of origin, gender, HLA-DRB1*15 and HLA-A*02. Allele groups within each gene were added to this baseline model to determine if any allele group had an additional independent effect. Within each model only those allele groups with a statistically significant odds ratio were kept in the next step in which a new model was created containing the baseline plus the significant allele groups from all genes. Allele groups were removed using stepwise regression until only significant ones were left. The model with the least number of included allele groups was compared to baseline. This analysis was performed twice with all allele groups coded as dose (0, 1, 2) or as carriage (0, 1) (in previous literature commonly referred to as “phenotype” referring to the co-dominant expression of HLA genes), with the exceptions being DRB1*15 which was previously seen to act multiplicatively and was therefore always coded as dose and A*02 which had a dominant effect in our cohort, hence always coded for carriage as described above. When stratifying the cohort into DRB1*15 positive or negative strata, all allele groups with a total count of less than 75 (2.5%) or 100 (2.6%) chromosomes, respectively, were grouped as an X allele within each gene (see Table S1). When stratifying for DRB1*15 status, A*19, B*15, C*03 and DRB1*04 were used as intercepts in the positive group and A*01, B*07, C*04 and DRB1*04 were used as intercept in the DRB1*15 negative group . DRB1*15 was coded as dose (i.e. 2, 1 or 0 alleles) in the DRB1*15 positive group, all other alleles were coded as carriage (i.e. 1 or 0). The significant allele groups did not change in either stratum when using the same intercept as in the full analysis (data not shown).

Allele group associations were also analyzed for interaction using deviation from multiplicativity of effects (i.e. significant interaction term in the logistic regression model) as well as using deviation from additivity of effects (estimated by attributable proportion (AP) due to interaction) as criteria for interaction [36], [37]. The deviation from multiplicativity of effects analysis was performed with 5 variables: country of origin, gender, DRB1*15 allele group carriage status, allele group carriage status and an interaction term between DRB1*15 and the allele group investigated. The individuals were coded as carriers or non carriers of the particular risk allele group as well as for DRB1*15. In the AP analyses, risk was defined as “not carrying the protective allele group” for those allele groups whose nominal effects were protective.

Haplotypes for individuals were estimated using UNPHASED 3.0.13 since this version of the program could handle larger number of alleles and the tolerance for convergence was set to 10^−6^ due to complexity of the matrix, only printing most likely haplotypes to file. Only individuals with a probability over 80% of carrying the assigned haplotypes were used in the subsequent analyses leaving2,886 individuals of 3,444 in the analysis and excluding 48 haplotypes out of 1,279.

There was no difference in allele group distribution in different age groups or between males and females (data not shown). Gender was included as a covariate anyway since gender is a risk factor for MS in itself. Country of origin was included as a covariate to exclude possible hidden effects from population stratification even though both populations were of Scandinavian origin.

The correlation between the genotyped SNP (rs4959039) and HLA-A allele groups was estimated with UNPHASED 3.0.13 as for the haplotype analysis, but the complexity did not require changing the tolerance for convergence in this case. To study the effect of the SNP on MS susceptibility, logistic regression was performed in R.

## Supporting Information

Table S1 Allele group frequencies in the DRB1*15 positive and DRB1*15 negative strata.
(DOC)

Table S2 Associations of each locus in regression analysis of all subjects (n = 3444). There is no difference in MS risk between carriage of only A*02 or both A*02 and C*05. All investigated allele groups except C*05 show robust association with MS. Country of origin and gender were included in the models as covariates
(DOC)

Table S3 Frequencies of estimated haplotypes, odds ratios and p-values from logistic regression with the 20 most common DRB1*15 carrying haplotypes.
(DOC)

Table S4 Frequencies of estimated haplotypes, odds ratios and p-values from logistic regression with the 20 most common A*02 carrying haplotypes.
(DOC)

Table S5 Frequencies of estimated haplotypes, odds ratios and p-values from logistic regression with the 20 most common B*12 carrying haplotypes.
(DOC)

Table S6 Frequencies of estimated haplotypes, odds ratios and p-values from logistic regression with the 20 most common C*05 carrying haplotypes.
(DOC)
