# Supplementary material for: Importance of Human Leukocyte Antigen (HLA) Class I and II Alleles on the Risk of Multiple Sclerosis
Source: PLoS One. 2012 May 7;7(5):e36779. doi: 10.1371/journal.pone.0036779 (PMC3346735; doi:10.1371/journal.pone.0036779)
Supplement: Table S1 — Allele group frequencies in the DRB1*15 positive and DRB1*15 negative strata. (DOC) [file pone.0036779.s001.doc]

**Table S1.** Allele group frequencies in the *DRB1*15* positive and *DRB1*15* negative strata.

|  | **Allele frequencies (%)** | | | | | |
| --- | --- | --- | --- | --- | --- | --- |
|  | **DRB1*15 positive** | | | **DRB1*15 negative** | | |
| **Allele group** | **Patients (N=2102)** | **Controls (N=952)** | **Total count less than 75** | **Patients (N=1466)** | **Controls (N=2368)** | **Total count less than 100** |
| **A*1** | 14.4 | 11.7 | No | 15.6 | 15.4 | no |
| **A*2** | 27.6 | 34.2 | No | 27.8 | 34.7 | no |
| **A*3** | 23.2 | 21.7 | No | 15.9 | 14.7 | no |
| **A*9** | 11.0 | 10.1 | No | 11.1 | 9.9 | no |
| **A*10** | 6.0 | 5.7 | No | 6.3 | 3.7 | no |
| **A*11** | 4.9 | 4.1 | No | 6.7 | 6.0 | no |
| **A*19** | 9.0 | 8.7 | No | 12.1 | 10.5 | no |
| **A*28** | 3.8 | 3.8 | No | 4.5 | 5.1 | no |
| **C*1** | 2.9 | 3.7 | No | 4.6 | 4.8 | no |
| **C*2** | 4.8 | 5.5 | No | 6.7 | 6.1 | no |
| **C*3** | 14.0 | 13.8 | No | 22.0 | 23.9 | no |
| **C*4** | 6.3 | 6.0 | No | 10.8 | 10.7 | no |
| **C*5** | 6.4 | 7.6 | No | 7.0 | 8.8 | no |
| **C*6** | 6.9 | 5.8 | No | 5.8 | 7.3 | no |
| **C*7** | 47.6 | 46.2 | No | 28.3 | 27.5 | no |
| **C*8** | 1.5 | 1.5 | Yes | 3.9 | 2.5 | no |
| **C*12** | 4.1 | 6.6 | No | 3.2 | 1.9 | yes |
| **C*14** | 0.6 | 0.4 | Yes | 1.9 | 1.2 | yes |
| **C*15** | 3.3 | 2.0 | No | 2.7 | 2.3 | yes |
| **C*16** | 1.2 | 0.9 | Yes | 1.9 | 2.2 | yes |
| **C*17** | 0.4 | 0.1 | Yes | 1.2 | 0.6 | yes |
| **C*18** | 0.0 | 0.0 | Yes | 0.1 | 0.0 | yes |
| **B*5** | 5.3 | 5.1 | No | 5.5 | 4.9 | no |
| **B*7** | 35.4 | 33.4 | No | 8.3 | 8.4 | no |
| **B*8** | 8.0 | 7.2 | No | 14.4 | 13.9 | no |
| **B*12** | 8.5 | 10.3 | No | 9.3 | 14.2 | no |
| **B*13** | 0.8 | 1.5 | Yes | 1.0 | 1.6 | yes |
| **B*14** | 1.9 | 0.9 | Yes | 4.0 | 2.2 | no |
| **B*15** | 8.8 | 8.3 | No | 14.5 | 13.1 | no |
| **B*16** | 1.5 | 2.4 | Yes | 3.0 | 2.0 | yes |
| **B*17** | 1.9 | 1.8 | Yes | 2.5 | 3.8 | no |
| **B*18** | 6.7 | 6.2 | No | 4.0 | 2.2 | no |
| **B*21** | 0.8 | 0.7 | Yes | 1.6 | 1.5 | yes |
| **B*22** | 1.0 | 1.6 | yes | 1.8 | 1.6 | yes |
| **B*27** | 4.9 | 6.2 | no | 7.0 | 7.6 | no |
| **B*35** | 5.2 | 5.3 | no | 9.7 | 8.8 | no |
| **B*37** | 3.1 | 2.2 | no | 1.6 | 1.2 | yes |
| **B*40** | 5.6 | 6.2 | no | 10.1 | 11.9 | no |
| **B*41** | 0.3 | 0.1 | yes | 1.2 | 0.5 | yes |
| **B*42** | 0.0 | 0.0 | yes | 0.0 | 0.0 | yes |
| **B*47** | 0.3 | 0.3 | yes | 0.4 | 0.4 | yes |
| **B*48** | 0.0 | 0.2 | yes | 0.1 | 0.1 | yes |
| **B*53** | 0.0 | 0.0 | yes | 0.0 | 0.0 | yes |
| **B*67** | 0.0 | 0.0 | yes | 0.0 | 0.0 | yes |
| **B*70** | 0.0 | 0.0 | yes | 0.0 | 0.0 | yes |
| **B*73** | 0.0 | 0.0 | yes | 0.1 | 0.0 | yes |
| **DRB1*1** | 3.6 | 6.3 | no | 13.6 | 14.2 | no |
| **DRB1*3** | 7.2 | 6.6 | no | 16.0 | 15.2 | no |
| **DRB1*4** | 10.8 | 11.6 | no | 23.2 | 23.2 | no |
| **DRB1*5** | 4.4 | 4.5 | no | 9.0 | 9.5 | no |
| **DRB1*6** | 5.4 | 7.8 | no | 20.6 | 19.2 | no |
| **DRB1*7** | 3.9 | 4.9 | no | 6.8 | 9.9 | no |
| **DRB1*8** | 3.5 | 2.9 | no | 7.0 | 5.9 | no |
| **DRB1*9** | 0.5 | 1.2 | yes | 1.5 | 1.5 | yes |
| **DRB1*10** | 0.4 | 0.5 | yes | 1.4 | 1.0 | yes |
| **DRB1*15** | 60.1 | 53.2 | no | 0.0 | 0.0 | - |
| **DRB1*16** | 0.3 | 0.5 | yes | 0.8 | 0.5 | yes |
