# Supplementary material for: Importance of Human Leukocyte Antigen (HLA) Class I and II Alleles on the Risk of Multiple Sclerosis
Source: PLoS One. 2012 May 7;7(5):e36779. doi: 10.1371/journal.pone.0036779 (PMC3346735; doi:10.1371/journal.pone.0036779)
Supplement: Table S2 — Associations of each locus in regression analysis of all subjects (n = 3444). There is no difference in MS risk between carriage of only A*02 or both A*02 and C*05. All investigated allele groups except C*05 show robust association with MS. Country of origin and gender were included in the models as covariates (DOC) [file pone.0036779.s002.doc]

**Table S2.** Associations of each locus in regression analysis of all subjects (n=3444). There is no difference in MS risk between carriage of only *A*02* or both *A*02* and *C*05*. All investigated allele groups except *C*05* show robust association with MS. Country of origin and gender were included in the models as covariates

|  | |  | |  | | Total population | | |  | | |  |  | | |  |  |  | | |  | |  | |
| --- | --- | --- | --- | --- | --- | --- | --- | --- | --- | --- | --- | --- | --- | --- | --- | --- | --- | --- | --- | --- | --- | --- | --- | --- |
| **Covariates, single markers** | | | | | ***HLA-A*02*** | | | | ***HLA-C*05*** | | | | ***HLA-B*12*** | | | | ***HLA-B*18*** | | | | ***Carry both HLA-A*02 and C*05*** | | | |
|  |  | |  | | ***OR*** | | ***p-value*** | | ***OR*** | ***p-value*** | | | ***OR*** | ***p-value*** | | | ***OR*** | | ***p-value*** | | ***OR*** | | | ***p-value*** |
| *DRB1*15* |  | |  | | 0.66 (0.58-0.76) | | 1.18x10^-08^ | | 0.77 (0.63-0.94) | 0.012 | | | 0.67 (0.56-0.80) | 6.16x10^-06^ | | | 1.39 (1.07-1.81) | | 0.013 | | 0.64 (0.50-0.82) | | | 2.87x10^-04^ |
| *DRB1*15* | *A*02* | |  | |  | | - | | 0.83 (0.68-1.02) | 0.1 | | | 0.70 (0.59-0.84) | 7.62x10^-05^ | | | 1.32 (1.02-1.72) | | 0.038 | |  | | | - |
| *DRB1*15* | *C*05* | |  | | 0.67 (0.58-0.78) | | 5.96x10^-08^ | |  | - | | | 0.65 (0.52-0.81) | 1.64x10^-04^ | | | 1.43 (1.10-1.86) | | 0.0074 | |  | | | - |
| *DRB1*15* | *B*12* | |  | | 0.68 (0.59-0.79) | | 1.40x10^-07^ | | 1.05 (0.81-1.36) | 0.7 | | |  | - | | |  | | - | | 0.82 (0.61-1.10) | | | 0.2 |
| *DRB1*15* | *B*18* | |  | | 0.67 (0.58-0.77) | | 3.05x10^-08^ | | 0.76 (0.62-0.93) | 0.0066 | | |  | - | | |  | | - | | 0.64 (0.50-0.82) | | | 3.03x10^-04^ |
| *DRB1*15* | *A*02* | | *C*05* | |  | | - | |  | - | | | 0.66 (0.53-0.83) | 2.96x10^-04^ | | | 1.35 (1.04-1.76) | | 0.025 | |  | | | - |
| *DRB1*15* | *A*02* | | *B*12* | |  | | - | | 1.12 (0.86-1.45) | 0.4 | | |  | - | | |  | | - | |  | | | - |
| *DRB1*15* | *A*02* | | *B*18* | |  | | - | | 0.81 (0.66-1.00) | 0.047 | | |  | - | | |  | | - | |  | | | - |
| *DRB1*15* | *C*05* | | *B*12* | | 0.68 (0.59-0.78) | | 1.06x10^-07^ | |  | - | | |  | - | | |  | | - | |  | | | - |
| *DRB1*15* | *C*05* | | *B*18* | | 0.68 (0.59-0.79) | | 1.81x10^-07^ | |  | - | | |  | - | | |  | | - | |  | | | - |
| **Covariate haplotypes, carriage of two markers** | | | | | ***DRB1*15*** | | | | ***HLA-B*12*** | | | | ***HLA-B*18*** | | | |  | | | | | | | |
|  | |  | |  | | ***OR*** | | ***p-value*** | ***OR*** | | ***p-value*** | | ***OR*** | | ***p-value*** | |  | | | | | | | |
| *A*02-C*05* | |  | |  | | 3.54 (3.07-4.09) | | 8.22x10^-71^ | 0.66 (0.54-0.81) | | 6.56x10^-05^ | | 1.75 (1.36-2.25) | | 8.15x10^-06^ | |  | | | | | | | |
| *A*02-C*05* | | *DRB1*15* | | |  | | | - | 0.72 (0.58-0.89) | | 0.0027 | | 1.39 (1.07-1.80) | | 0.014 | |  | | |  | |  | |  |
