# Supplementary material for: Importance of Human Leukocyte Antigen (HLA) Class I and II Alleles on the Risk of Multiple Sclerosis
Source: PLoS One. 2012 May 7;7(5):e36779. doi: 10.1371/journal.pone.0036779 (PMC3346735; doi:10.1371/journal.pone.0036779)
Supplement: Table S3 — Frequencies of estimated haplotypes, odds ratios and p-values from logistic regression with the 20 most common DRB1*15 carrying haplotypes. (DOC) [file pone.0036779.s003.doc]

**Table S3. Frequencies of estimated haplotypes, odds ratios and p-values from logistic regression with the 20 most common *DRB1*15* carrying haplotypes.**

|  | **Haplotype, DRB1*15 positive** | | | |  |  |  |  |  |  |  |  |
| --- | --- | --- | --- | --- | --- | --- | --- | --- | --- | --- | --- | --- |
| **No.** | **HLA-A** | **HLA-C** | **HLA-B** | **HLA-DRB1** | **Cases** | **Controls** | **Frequency Cases (%)** | **Frequency Controls (%)** | **Frequency Total (%)** | **Nominal p-value** | **FDR corrected p-values** | **Odds Ratio (95% CI)** |
| **1.** | 3 | 7 | 7 | 15 | 226 | 118 | 7.6 | 4.2 | 6.0 | 8.21x10^-11^ | 6.30x10^-10^ | 2.24 (1.76-2.86) |
| **2.** | 2 | 7 | 7 | 15 | 179 | 59 | 6.0 | 2.1 | 4.1 | 2.15x10^-14^ | 4.95x10^-13^ | 3.40 (2.50-4.69) |
| **3.** | 9 | 7 | 7 | 15 | 78 | 23 | 2.6 | 0.8 | 1.7 | 1.75x10^-07^ | 1.00x10^-06^ | 3.63 (2.27-6.01) |
| **4.** | 10 | 12 | 18 | 15 | 44 | 22 | 1.5 | 0.8 | 1.1 | 0.0017 | 0.0057 | 2.36 (1.40-4.12) |
| **5.** | 1 | 6 | 37 | 15 | 37 | 10 | 1.2 | 0.4 | 0.8 | 3.77x10^-05^ | 0.00017 | 4.45 (2.27-9.54) |
| **6.** | 2 | 5 | 12 | 15 | 24 | 23 | 0.8 | 0.8 | 0.8 | 0.68 | 0.71 | 1.13 (0.62-2.07) |
| **7.** | 1 | 7 | 7 | 15 | 30 | 15 | 1.0 | 0.5 | 0.8 | 0.011 | 0.023 | 2.30 (1.23-4.46) |
| **8.** | 1 | 7 | 8 | 15 | 29 | 0 | 1.0 | 0.0 | 0.5 | 0.95 | 0.95 | 28.79 (8.22-101)* |
| **9.** | 2 | 3 | 15 | 15 | 19 | 8 | 0.6 | 0.3 | 0.5 | 0.031 | 0.051 | 2.57 (1.12-6.39) |
| **10.** | 19 | 7 | 7 | 15 | 16 | 4 | 0.5 | 0.1 | 0.3 | 0.0068 | 0.017 | 4.65 (1.67-16.4) |
| **11.** | 11 | 7 | 7 | 15 | 13 | 5 | 0.4 | 0.2 | 0.3 | 0.1 | 0.14 | 2.47 (0.89-7.94) |
| **12.** | 3 | 4 | 35 | 15 | 17 | 1 | 0.6 | 0.0 | 0.3 | 0.0027 | 0.0076 | 22.39 (4.53-407) |
| **13.** | 19 | 15 | 5 | 15 | 11 | 6 | 0.4 | 0.2 | 0.3 | 0.092 | 0.14 | 2.38 (0.89-6.97) |
| **14.** | 3 | 7 | 18 | 15 | 11 | 6 | 0.4 | 0.2 | 0.3 | 0.13 | 0.18 | 2.19 (0.81-6.47) |
| **15.** | 2 | 2 | 27 | 15 | 8 | 6 | 0.3 | 0.2 | 0.2 | 0.38 | 0.44 | 1.62 (0.55-4.97) |
| **16.** | 9 | 2 | 27 | 15 | 11 | 3 | 0.4 | 0.1 | 0.2 | 0.025 | 0.048 | 4.43 (1.34-19.9) |
| **17.** | 10 | 7 | 7 | 15 | 5 | 8 | 0.2 | 0.3 | 0.2 | 0.27 | 0.34 | 0.49 (0.13-1.63) |
| **18.** | 19 | 7 | 18 | 15 | 7 | 5 | 0.2 | 0.2 | 0.2 | 0.53 | 0.58 | 1.47 (0.45-5.11) |
| **19.** | 2 | 3 | 40 | 15 | 7 | 3 | 0.2 | 0.1 | 0.2 | 0.31 | 0.37 | 2.07 (0.55-9.91) |
| **20.** | 2 | 7 | 12 | 15 | 9 | 1 | 0.3 | 0.0 | 0.2 | 0.03 | 0.051 | 10.21 (1.84-191) |

*= Odds ratio manually calculated as in Haldane JB et al [38] to correct for missing values.
