# Supplementary material for: Importance of Human Leukocyte Antigen (HLA) Class I and II Alleles on the Risk of Multiple Sclerosis
Source: PLoS One. 2012 May 7;7(5):e36779. doi: 10.1371/journal.pone.0036779 (PMC3346735; doi:10.1371/journal.pone.0036779)
Supplement: Table S4 — Frequencies of estimated haplotypes, odds ratios and p-values from logistic regression with the 20 most common A*02 carrying haplotypes. (DOC) [file pone.0036779.s004.doc]

**Table S4. Frequencies of estimated haplotypes, odds ratios and p-values from logistic regression with the 20 most common** ***A*02* carrying haplotypes.**

|  | **Haplotype, A*02 positive** | | | |  |  |  |  |  |  |  |  |
| --- | --- | --- | --- | --- | --- | --- | --- | --- | --- | --- | --- | --- |
| **No.** | **HLA-A** | **HLA-C** | **HLA-B** | **HLA-DRB1** | **Cases** | **Controls** | **Frequency Cases (%)** | **Frequency Controls (%)** | **Frequency Total (%)** | **Nominal p-value** | **FDR corrected p-values** | **Odds Ratio (95% CI)** |
| **1.** | 2 | 7 | 7 | 15 | 179 | 59 | 6.0 | 2.1 | 4.1 | 1.67x10^-10^ | 3.84x10^-09^ | 2.79 (2.05-3.85) |
| **2.** | 2 | 3 | 15 | 4 | 53 | 86 | 1.8 | 3.1 | 2.4 | 8.70x10^-04^ | 4.60x10^-03^ | 0.54 (0.37-0.77) |
| **3.** | 2 | 5 | 12 | 4 | 25 | 81 | 0.8 | 2.9 | 1.8 | 8.86x10^-09^ | 1.02x10^-07^ | 0.26 (0.16-0.40) |
| **4.** | 2 | 3 | 40 | 6 | 38 | 56 | 1.3 | 2.0 | 1.6 | 0.012 | 0.025 | 0.57 (0.37-0.88) |
| **5.** | 2 | 5 | 12 | 15 | 24 | 23 | 0.8 | 0.8 | 0.8 | 0.75 | 0.78 | 0.91 (0.50-1.66) |
| **6.** | 2 | 7 | 8 | 3 | 16 | 26 | 0.5 | 0.9 | 0.7 | 0.037 | 0.061 | 0.50 (0.26-0.95) |
| **7.** | 2 | 3 | 40 | 4 | 17 | 24 | 0.6 | 0.9 | 0.7 | 0.16 | 0.021 | 0.63 (0.32-1.20) |
| **8.** | 2 | 3 | 15 | 6 | 16 | 24 | 0.5 | 0.9 | 0.7 | 0.13 | 0.17 | 0.61 (0.31-1.15) |
| **9.** | 2 | 2 | 27 | 4 | 9 | 23 | 0.3 | 0.8 | 0.6 | 0.0074 | 0.021 | 0.34 (0.15-0.73) |
| **10.** | 2 | 7 | 7 | 6 | 13 | 16 | 0.4 | 0.6 | 0.5 | 0.32 | 0.39 | 0.68 (0.32-1.44) |
| **11.** | 2 | 6 | 17 | 7 | 8 | 20 | 0.3 | 0.7 | 0.5 | 0.020 | 0.035 | 0.37 (0.15-0.82) |
| **12.** | 2 | 3 | 15 | 15 | 19 | 8 | 0.6 | 0.3 | 0.5 | 0.10 | 0.15 | 2.01 (0.89-4.94) |
| **13.** | 2 | 4 | 35 | 6 | 6 | 20 | 0.2 | 0.7 | 0.5 | 0.0082 | 0.021 | 0.28 (0.10-0.68) |
| **14.** | 2 | 3 | 40 | 8 | 8 | 14 | 0.3 | 0.5 | 0.4 | 0.34 | 0.39 | 0.64 (0.24-1.59) |
| **15.** | 2 | 6 | 13 | 7 | 3 | 19 | 0.1 | 0.7 | 0.4 | 0.0032 | 0.012 | 0.15 (0.04-0.47) |
| **16.** | 2 | 1 | 27 | 1 | 2 | 18 | 0.1 | 0.6 | 0.3 | 0.0010 | 0.0046 | 0.08 (0.01-0.30) |
| **17.** | 2 | 2 | 27 | 8 | 10 | 9 | 0.3 | 0.3 | 0.3 | 0.54 | 0.60 | 0.74 (0.27-1.97) |
| **18.** | 2 | 5 | 12 | 5 | 4 | 14 | 0.1 | 0.5 | 0.3 | 0.013 | 0.025 | 0.24 (0.07-0.68) |
| **19.** | 2 | 5 | 12 | 6 | 2 | 16 | 0.1 | 0.6 | 0.3 | 0.0038 | 0.013 | 0.11 (0.02-0.40) |
| **20.** | 2 | 3 | 40 | 1 | 0 | 17 | 0.0 | 0.6 | 0.3 | 0.94 | 0.94 | 0.05 (0.013-0.20)* |

*= Odds ratio manually calculated as in Haldane JB et al [38] to correct for missing values.
