# Supplementary material for: Importance of Human Leukocyte Antigen (HLA) Class I and II Alleles on the Risk of Multiple Sclerosis
Source: PLoS One. 2012 May 7;7(5):e36779. doi: 10.1371/journal.pone.0036779 (PMC3346735; doi:10.1371/journal.pone.0036779)
Supplement: Table S5 — Frequencies of estimated haplotypes, odds ratios and p-values from logistic regression with the 20 most common B*12 carrying haplotypes. (DOC) [file pone.0036779.s005.doc]

**Table S5.** Frequencies of estimated haplotypes, odds ratios and p-values from logistic regression with the 20 most common *B*12* carrying haplotypes.

|  | **Haplotype, B*12 positive** | | | |  |  |  |  |  |  |  |  |
| --- | --- | --- | --- | --- | --- | --- | --- | --- | --- | --- | --- | --- |
| **No.** | **HLA-A** | **HLA-C** | **HLA-B** | **HLA-DRB1** | **Cases** | **Controls** | **Frequency Cases (%)** | **Frequency Controls (%)** | **Frequency Total (%)** | **Nominal p-value** | **FDR corrected p-values** | **Odds Ratio (95% CI)** |
| **1.** | 2 | 5 | 12 | 4 | 25 | 81 | 0.84 | 2.89 | 1.84 | 5.26x10^-08^ | 1.21x10^-06^ | 0.28 (0.17-0.44) |
| **2.** | 2 | 5 | 12 | 15 | 24 | 23 | 0.81 | 0.82 | 0.81 | 0.72 | 0.75 | 0.90 (0.49-1.64) |
| **3.** | 19 | 16 | 12 | 7 | 21 | 22 | 0.71 | 0.79 | 0.74 | 0.70 | 0.75 | 0.89 (0.48-1.64) |
| **4.** | 9 | 4 | 12 | 7 | 12 | 17 | 0.40 | 0.61 | 0.50 | 0.28 | 0.43 | 0.66 (0.31-1.39) |
| **5.** | 19 | 5 | 12 | 5 | 7 | 12 | 0.24 | 0.43 | 0.33 | 0.20 | 0.36 | 0.54 (0.20-1.36) |
| **6.** | 2 | 5 | 12 | 5 | 4 | 14 | 0.13 | 0.50 | 0.31 | 0.01 | 0.044 | 0.23 (0.06-0.64) |
| **7.** | 2 | 5 | 12 | 6 | 2 | 16 | 0.07 | 0.57 | 0.31 | 0.0037 | 0.028 | 0.11 (0.02-0.40) |
| **8.** | 2 | 5 | 12 | 1 | 9 | 6 | 0.30 | 0.21 | 0.26 | 0.50 | 0.60 | 1.44 (0.51-4.44) |
| **9.** | 28 | 7 | 12 | 5 | 6 | 9 | 0.20 | 0.32 | 0.26 | 0.26 | 0.42 | 0.55 (0.18-1.53) |
| **10.** | 19 | 5 | 12 | 4 | 4 | 9 | 0.13 | 0.32 | 0.23 | 0.11 | 0.26 | 0.39 (0.10-1.19) |
| **11.** | 2 | 16 | 12 | 7 | 4 | 7 | 0.13 | 0.25 | 0.19 | 0.32 | 0.46 | 0.53 (0.14-1.78) |
| **12.** | 2 | 7 | 12 | 15 | 9 | 1 | 0.30 | 0.04 | 0.17 | 0.065 | 0.18 | 7.11 (1.29-132) |
| **13.** | 2 | 7 | 12 | 5 | 3 | 7 | 0.10 | 0.25 | 0.17 | 0.13 | 0.26 | 0.35 (0.07-1.27) |
| **14.** | 28 | 7 | 12 | 1 | 4 | 6 | 0.13 | 0.21 | 0.17 | 0.41 | 0.53 | 0.58 (0.15-2.09) |
| **15.** | 3 | 5 | 12 | 6 | 8 | 1 | 0.27 | 0.04 | 0.16 | 0.064 | 0.18 | 7.18 (1.31-134) |
| **16.** | 11 | 5 | 12 | 5 | 1 | 6 | 0.03 | 0.21 | 0.12 | 0.069 | 0.18 | 0.14 (0.01-0.83) |
| **17.** | 19 | 5 | 12 | 15 | 7 | 0 | 0.24 | 0.00 | 0.12 | 0.95 | 0.95 | 7.56 (1.62-35.4)* |
| **18.** | 19 | 16 | 12 | 5 | 2 | 4 | 0.07 | 0.14 | 0.10 | 0.39 | 0.53 | 0.47 (0.06-2.5) |
| **19.** | 19 | 6 | 12 | 4 | 2 | 4 | 0.07 | 0.14 | 0.10 | 0.52 | 0.60 | 0.57 (0.08-3.05) |
| **20.** | 2 | 5 | 12 | 7 | 1 | 5 | 0.03 | 0.18 | 0.10 | 0.14 | 0.26 | 0.19 (0.01-1.24) |

*= Odds ratio manually calculated as in Haldane JB et al [38] to correct for missing values.
