# Supplementary material for: Importance of Human Leukocyte Antigen (HLA) Class I and II Alleles on the Risk of Multiple Sclerosis
Source: PLoS One. 2012 May 7;7(5):e36779. doi: 10.1371/journal.pone.0036779 (PMC3346735; doi:10.1371/journal.pone.0036779)
Supplement: Table S6 — Frequencies of estimated haplotypes, odds ratios and p-values from logistic regression with the 20 most common C*05 carrying haplotypes. (DOC) [file pone.0036779.s006.doc]

**Table S6. Frequencies of estimated haplotypes, odds ratios and p-values from logistic regression with the 20 most common *C*05* carrying haplotypes.**

|  | **Haplotype, C*05 positive** | | | |  |  |  |  |  |  |  |  |
| --- | --- | --- | --- | --- | --- | --- | --- | --- | --- | --- | --- | --- |
| **No.** | **HLA-A** | **HLA-C** | **HLA-B** | **HLA-DRB1** | **Cases** | **Controls** | **Frequency Cases (%)** | **Frequency Controls (%)** | **Frequency Total (%)** | **Nominal p-value** | **FDR corrected p-values** | **Odds Ratio (95% CI)** |
| **1.** | 2 | 5 | 12 | 4 | 25 | 81 | 0.84 | 2.89 | 1.84 | 6.70x10^-08^ | 1.54x10^-06^ | 0.28 (0.18-0.44) |
| **2.** | 2 | 5 | 12 | 15 | 24 | 23 | 0.81 | 0.82 | 0.81 | 0.72 | 0.88 | 0.90 (0.49-1.64) |
| **3.** | 19 | 5 | 12 | 5 | 7 | 12 | 0.24 | 0.43 | 0.33 | 0.19 | 0.33 | 0.53 (0.20-1.33) |
| **4.** | 2 | 5 | 12 | 5 | 4 | 14 | 0.13 | 0.50 | 0.31 | 0.010 | 0.048 | 0.23 (0.07-0.65) |
| **5.** | 2 | 5 | 12 | 6 | 2 | 16 | 0.07 | 0.57 | 0.31 | 0.0039 | 0.024 | 0.11 (0.02-0.40) |
| **6.** | 2 | 5 | 12 | 1 | 9 | 6 | 0.30 | 0.21 | 0.26 | 0.49 | 0.67 | 1.45 (0.51-4.46) |
| **7.** | 19 | 5 | 12 | 4 | 4 | 9 | 0.13 | 0.32 | 0.23 | 0.12 | 0.27 | 0.39 (0.11-1.21) |
| **8.** | 19 | 5 | 18 | 3 | 10 | 3 | 0.34 | 0.11 | 0.23 | 0.13 | 0.27 | 2.72 (0.83-12.19) |
| **9.** | 3 | 5 | 12 | 6 | 8 | 1 | 0.27 | 0.04 | 0.16 | 0.062 | 0.20 | 7.30 (1.33-136) |
| **10.** | 11 | 5 | 12 | 5 | 1 | 6 | 0.03 | 0.21 | 0.12 | 0.072 | 0.21 | 0.14 (0.01-0.84) |
| **11.** | 19 | 5 | 12 | 15 | 7 | 0 | 0.24 | 0.00 | 0.12 | 0.97 | 0.97 | 7.56 (1.62-35.4)* |
| **12.** | 2 | 5 | 18 | 3 | 6 | 1 | 0.20 | 0.04 | 0.12 | 0.13 | 0.27 | 5.11 (0.86-97.2) |
| **13.** | 28 | 5 | 5 | 6 | 0 | 7 | 0.00 | 0.25 | 0.12 | 0.96 | 0.97 | 0.12 (0.026-0.55)* |
| **14.** | 2 | 5 | 12 | 7 | 1 | 5 | 0.03 | 0.18 | 0.10 | 0.14 | 0.27 | 0.19 (0.01-1.26) |
| **15.** | 28 | 5 | 18 | 3 | 4 | 2 | 0.13 | 0.07 | 0.10 | 0.66 | 0.84 | 1.48 (0.27-11.0) |
| **16.** | 1 | 5 | 12 | 15 | 5 | 0 | 0.17 | 0.00 | 0.09 | 0.97 | 0.97 | 5.66 (1.18-27.1)* |
| **17.** | 11 | 5 | 12 | 7 | 1 | 3 | 0.03 | 0.11 | 0.07 | 0.34 | 0.53 | 0.33 (0.02-2.65) |
| **18.** | 19 | 5 | 5 | 6 | 3 | 1 | 0.10 | 0.04 | 0.07 | 0.45 | 0.64 | 2.45 (0.29-50.7) |
| **19.** | 3 | 5 | 12 | 5 | 0 | 4 | 0.00 | 0.14 | 0.07 | 0.97 | 0.97 | 0.19 (0.040-0.91)* |
| **20.** | 9 | 5 | 12 | 4 | 1 | 3 | 0.03 | 0.11 | 0.07 | 0.22 | 0.37 | 0.24 (0.01-1.93) |

*= Odds ratio manually calculated as in Haldane JB et al [38] to correct for missing values.
